# Langerhans cells prevent subbasal nerve damage and upregulate neurotrophic factors in dry eye disease

**DOI:** 10.1371/journal.pone.0176153

**Published:** 2017-04-25

**Authors:** Eun Young Choi, Hyun Goo Kang, Chul Hee Lee, Areum Yeo, Hye Mi Noh, Nayeong Gu, Myoung Joon Kim, Jong Suk Song, Hyeon Chang Kim, Hyung Keun Lee

**Affiliations:** 1 Department of Ophthalmology, Yonsei University College of Medicine, Seoul, Republic of Korea; 2 Department of Ophthalmology, Asan Medical Center, University of Ulsan College of Medicine, Seoul, Republic of Korea; 3 Department of Ophthalmology, Korea University College of Medicine, Seoul, Republic of Korea; 4 Department of Preventive Medicine and Public Health, Yonsei University College of Medicine, Seoul, Republic of Korea; Wayne State University School of Medicine, UNITED STATES

## Abstract

The functional role of Langerhans cells (LCs) in ocular surface inflammation and nerve damage in dry eye (DE) disease has yet to be determined. This study was performed to investigate this relationship through both clinical study on DE patients and in vivo mouse models with induced DE disease. In a cross-sectional case-control study (54 eyes of DE patients; 34 eyes of control patients), average cell density, area, and process length of LCs were measured using confocal microscopy. Data were analyzed to determine whether changes in LCs are correlated with subbasal nerve plexus (SNP) parameters (nerve density, beading, and tortuosity). In DE patients, SNP density marginally decreased and nerve beading and tortuosity were significantly increased compared to the control group. The total number of LCs significantly increased in DE patients, and some LCs with elongated processes were found to be attached to nerve fibers. Interestingly, nerve loss and deformation were correlated with inactivation of LCs. In an *in vivo* experiment to elucidate the role of LCs in ocular surface inflammation and corneal nerve loss, we used a genetically modified mouse model (CD207-DTR) that reduced the population of CD207 (Langerin) expressing cells by injection of diphtheria toxin. In CD207-depleted mice with DE disease (CD207-dDTR+DE), corneal nerves in the central region were significantly decreased, an effect that was not observed in wild-type (WT)+DE mice. In CD207-dDTR+DE mice, infiltration of CD4+, CD19+, CD45+, and CD11b+ cells into the ocular surface was increased, as confirmed by flow cytometry. Increased IL-17 and IFN-γ mRNA levels, and decreased expression of neurotrophic factors and neurotransmitters, were also found in the CD207-dDTR+DE mice. These data support a functional role for LCs in negatively regulating ocular surface inflammation and exhibiting a neuroprotective function in DE disease.

## Introduction

Although the precise pathophysiology of dry eye (DE) disease is unknown, immuno-inflammatory responses [1,2] and the loss of neural regulation between ocular surface and the lacrimal gland [3,4] are considered to be important contributing factors in DE initiation and progression. It has been shown extensively that, for both humans and mice, activation and recruitment of CD11c^+^ and CD11b^+^ antigen-presenting cells (APCs) [5] increase Th1 and Th17 T cell infiltration [6], which in turn upregulates chemokines and inflammatory cytokines on the ocular surface [7–9]. Likewise, numerous reports indicate that corneal nerve changes are associated with DE disease. Loss of the corneal subbasal nerve plexus (SNP), reduced nerve leashes and branching, and increased nerve fiber beading have been noted as typical changes on the surface of eyes with DE [10,11]. Moreover, neurotrophic factors (known stimulators of nerve regeneration) have been reported to be increased in the tears of DE patients [12,13].

Langerhans cells (LCs), a subtype of dendritic cells (DCs), are the most potent antigen-presenting cells found in the epithelial layer. They are distinguishable from other DCs by their expression of Langerin or CD207 (a C-type lecitin) and Birbeck granules [14]. Like the skin [15], the ocular surface also contains DCs including LCs (which express CD207) and other DCs (which express CD11c and/or CD11b). Recently, Hattori et al. reported that the corneal epithelium contains LCs expressing Langerin and CD11c markers, which may be separated from Langerin+ non-LCs in the stroma by *ex vivo* examination[16]. In previous studies, it was shown that CD11c+ cells are recruited and activated in DE, modulating immune response in the ocular surface [1,17]. In humans, *in vivo* confocal microscopy (IVCM) has been used in several studies to describe the morphological and population changes of intraepithelial DCs (considered LCs in the DE disease affected cornea) [18,19], and an increased number of LCs with elongated processes have been found in both non-Sjogren’s syndrome and Sjogren’s syndrome patients [18]. However, there are few studies investigating the function of CD207+ cells (represented by LCs) in DE-induced pathogenesis.

The neuroimmune connection has been proposed by a growing body of studies in the skin [20,21]. It is supposed that representative inflammatory skin diseases (e.g. psoriasis [22], atopic dermatitis [23], rosacea [24], and acne [25]) are exacerbated by nervous stimulation. Moreover, considerable amount of evidence indicate that not only nerves are essential in the migration and activation of DCs [26,27], but also DCs play an important role in maintaining sensory nerves [28,29]. Therefore, it may be assumed that the interaction between the immune system (represented by LCs) and neural networks is an important factor in DE pathophysiology; however, there have been no specific reports describing this interaction.

Two studies were conducted to investigate the role of the inflammatory response and the nervous system in DE disease. A case-control clinical study was performed to investigate possible correlations between corneal LC changes and subbasal nerve loss in DE patients. To elucidate the role of LCs in DE focusing on changes of the ocular surface inflammatory responses and neural integrity, we induced DE in the CD207-DTR mice, which lack CD207+ cells in the epidermis, including the ocular surface.

## Materials and methods

Studies were conducted at two independent sites. The cross-sectional, case-control clinical study was conducted at the Department of Ophthalmology, Yonsei University College of Medicine, Seoul, Korea. The animal (mouse) model DE studies were performed at the Clinical Research Center of the same institute.

### Clinical study

#### Study population

A total of fifty-four eyes from 44 non-Sjogren DE patients (19 males and 25 females) with a mean age ± standard deviation (SD) of 49.3 ± 12.5 years (range 22–78 years) was used in this study. Patients included those who had experienced DE disease-related symptoms (such as dryness, foreign body sensation, or irritation) for more than 6 months with 1) a sign of superficial punctate erosion of conjunctiva and/or cornea in relevant eye, 2) a Schirmer test result (with anesthesia) of 8 mm in 5 minutes or less, or 3) a tear film break-up time of 5 seconds or less. Exclusion criteria were 1) the use of any anti-inflammatory eye drops in the 3 months preceding the study, 2) a history of ocular infection, trauma, or surgery in the 6 months preceding the study, 3) meibomian gland dysfunction of stage 3 or more (per the criteria proposed by the International Workshop on MGD 2011 [30]), or blinking abnormalities, 4) an uncontrolled systemic disease, or 5) current pregnancy and/or lactation status.

The control group was comprised of 34 eyes from 17 age- and sex-matched subjects (6 males and 11 females) who had no history of ocular dryness, with clear cornea, a tear film break-up time of more than 8 seconds, and a Schirmer test value of more than 10 mm for a duration of 5 minutes. The mean age of control subjects was 52.9 ± 22.3 years (range 24–77 years). All procedures conformed to the tenants of the Declaration of Helsinki, and informed consent was obtained from all patients after the Institutional Review Board (IRB)/Ethics Committee of Severance Medical Center approval was obtained. A written informed consent was obtained before the examination from each patient.

#### IVCM of intraepithelial DCs and SNP

All patients were examined with a confocal laser-scanning microscope (HRT II RCM, Rostock Corneal Module, Heidelberg Engineering, Heidelberg, Germany), equipped with Heidelberg Eye Explorer, version 1.5.10.0 software (Heidelberg Engineering). During confocal examination in sequence mode, a series of confocal images was recorded by two masked observers as the focal plane manually advanced from anterior to the posterior of the endothelium. Each image represented a corneal section approximately 400 × 400 μm (horizontal × vertical) with a lateral spatial resolution of 0.5 μm and a depth resolution of 1.2 μm. Four to eight complete scans were recorded in each of two camera modes: fixed gain (constant gain, voltage, and black level) and automatic gain (the gain, voltage, and black level varied throughout image acquisition).

All confocal scans with either intraepithelial DC (defined as LC) or SNP visualization at the center of the cornea were selected. Among them, the five most well focused images were analyzed by two masked observers. LCs were designated as hyper-reflective cellar bodies with or without dendrites, located in the basal epithelial layer. After manually marking them in each frame, the LC densities (cells/mm^2^) were automatically calculated with ImageJ software (Version 1.47, NIH, Maryland, USA). Average length of processes (μm) and mean cell area (μm^2^) of six to eight LCs were measured using the Freehand Line and Measure Area tools of the software in magnified images (x200). The SNP appeared as long, narrow structures between the basal epithelial layer and the Bowman layer in the selected images. Total length of the nerves within a frame was calculated as nerve density (μm/mm^2^). The Freehand Line tool was also used to trace along nerve fibers in addition to the NeuronJ plug-in for ImageJ. The number of beading was counted on all nerve fibers in a frame and indicated as nerve beading [31]. Nerve tortuosity was graded from 0 to 4 following the Oliveira-Soto and Enfron method [32].

### Experimental study

#### Mouse models and DE induction

Male, 6- to 8-week old C57BL/6 wild-type (WT) mice (Charles River Laboratory, Wilmington, MA, USA) and Langerin/CD207-diphtheria toxin receptor (CD207-DTR) B6.129S2-Cd207tm3(DTR/GFP)Mal/J mice (Jackson Laboratory, Bar Harbor, ME, USA) were obtained for the study and used in accordance with the standards of the Association for Research in Vision and Ophthalmology (ARVO) Statement for the Use of Animals in Ophthalmic and Visual Research. The research protocol was approved by the Yonsei University Health System Institutional Animal Care and Use Committee (Permit number: LML 11–18). The health of animals was monitored by daily basis, and a trained person checked for signs of illness, injury, or abnormal behavior. In this study, none of the animals became severely ill nor died prior to the experimental endpoint. We had a protocol of early euthanasia for the animals who become severely ill during the experiments to relieve distress and pain. Mice were judged to be severely ill when they showed one or more of the following clinical signs over 1 week: weight loss, eating less, fecal shape changes, loss of hair quality or skin turgor, sluggish movement, a hunched posture. The protocol for early euthanasia was as follows. First, mice were placed in the gradual-fill CO2 chamber with a 15%/min displacement rate according to the American Veterinary Medical Association Guidelines for the Euthanasia of Animals: 2013 Edition. Cervical dislocation was immediately performed after complete loss of consciousness. All procedures in the experiment (e.g. corneal erosion grading) were performed under the anesthesia with Tiletamine-Zolazepam (40mg/kg) and Xylazine (5mg/kg) and all efforts were made to minimize suffering. At the endpoint of the experiment, mice were placed in the CO2 euthanasia chamber. Once mice were fully anesthetized, neck dislocation was performed following the tissue collection to confirm death by qualified individuals.

For the CD207-depleted DTR (CD207-dDTR) mice, 1.0 μg of diphtheria toxin was injected every 2 days for a period of 10 days (S1 Fig) to completely remove all CD207 DCs, including LCs. For each experiment, the removal of CD207+ cells was confirmed by flow cytometry. To exclude the possibility that diphtheria toxin may be cytotoxic or have effects of LC depletion independently, we injected the same dosage of saline (n = 2) and diphtheria toxin (n = 2) to WT mice.

Dry eye was induced (S1 Fig) by placing the mice in an environment-controlled chamber as described formerly [33]. To achieve maximum ocular surface dryness in the dry chamber, the mice were subcutaneously injected with 0.1 mL of 5 mg/mL scopolamine hydrobromide (Sigma-Aldrich, St. Louis, MO, USA), three times per day. Standard desiccating stress induction was done for 10 days. Before and during DE induction (day 1, day 3, and day 7), corneal erosion was graded with fluorescein staining accordance with Oxford scheme [34].

The eyeballs, skin, and lung tissues were harvested for analysis. The corneas and adjacent conjunctivas were separated from the eyeballs. The ocular surface tissues were divided into three and each part was prepared for immunofluorescence staining, flow cytometry analysis, and quantitative real-time polymerase chain reaction analysis (qRT-PCR). The lung tissues were grinded up with a homogenizer without preprocessing. Full-thickness skin tissues were separated from fat and connective tissues. After fragmentation, the samples were incubated in 0.25% trypsin EDTA overnight at 4°C and epidermal cells were scrapped out.

#### Immunofluorescence imaging of whole-mount corneas

Four whole-mount corneas from each group (WT and CD207-DTR mice without DE disease, CD207-DTR+DE mice, and CD207-dDTR+DE mice) were dissected into quadrants and fixed in 4% paraformaldehyde fixative (PFA) in phosphate-buffered saline (PBS) for 45 minutes. After two one-hour washes in PBS, corneas were blocked and permeabilized in blocking solution (PBS containing 2.0% bovine serum albumin [BSA]) for one hour shaking at room temperature. The cornea samples were incubated in diluted primary antibody (pRb a-Ms β-III tubulin ab18207, Abcam, Cambridge, MA, USA) overnight at 37°C. Corneas were then washed in PBS for one hour, and then washed again. Diluted secondary antibody (FITC anti-rabbit #406403, Biolegend, San Diego, CA, USA) was applied to the corneas with overnight shaking at 4°C. After two one-hour washes in PBS, corneas were mounted on glass slides with mounting medium containing fluorescence (Vectashield, Burlingame, CA, USA). A light microscope (Axio Imager 2, Carl Zeiss, Oberkochen, Germany) was used to visualize specimens. Epifluorescence images at 50× and 200× were obtained using Axiovision Rel. 4.8 software (Carl Zeiss). Images were compiled and analyzed using ImageJ to compare nerve density between the limbal area and cornea.

#### Measurement of ocular surface proinflammatory cytokines, neurotrophic factors, and neurotransmitters

Four to six corneas and adjunct conjunctivas from two to three mice were used in each group (WT and CD207-DTR mice without DE disease, CD207-DTR+DE mice, and CD207-dDTR+DE mice). Each experiment was performed in triplicate. RNA from mouse corneas and conjunctivas was isolated with the RNeasy Micro Kit (Qiagen, Hilden, Germany) and reverse transcribed using the Superscript III Kit (Invitrogen, Carlsbad, CA). qRT-PCR was performed using Taq-Man Universal PCR Mastermix (Applied Biosystems, Foster City, CA, USA) and preformulated primers (see S1 Table for detailed primer information) in StepOnePlus RT-PCR System (Applied Biosystems). The results were derived by the comparative threshold cycle method and normalized using GAPDH as a control.

#### Cell sorting and flow cytometry

As previously described [35], single-cell suspensions of corneal samples were prepared by collagenase digestion and blocked with anti-FcR mAb for 30 minutes at 4°C in 1% BSA in PBS. The isolated cells were stained with the following antibodies: anti-CD11b APC, anti-CD45 PE, anti-CD19 PE/cy7, and anti-CD4 FITC (Biolegend). All antibodies were analyzed with appropriate isotype controls. Cells were analyzed using FACSCanto and FACSAria flow cytometers (BD Biosciences, San Jose, CA, USA). Additional cornea and conjunctiva samples were prepared for intracellular cytokine staining with anti-CD207 APC, anti-IFN-γ FITC, and anti-IL-17 PE/cy7 (BioLegend) according to the manufacturer’s instructions.

### Statistical analysis

The Statistical Package for the Social Sciences (SPSS version 13.0; IBM, Chicago, IL, USA) was used for data analysis. Data were expressed as mean ± standard deviation (SD) for all variables. A value of p < 0.05 indicated statistically significant results and all statistical tests were 2-sided and used a 95% confidence interval.

Of the 88 eyes of 61 subjects enrolled in the study, only one eye in each patient was used for statistical analysis. Each variable of healthy control and DE patients were compared by using Fisher’s exact test for frequency data, Student’s t-test for continuous data, and the Mann–Whitney U test for non-normally distributed data. The association between variables was examined using the Pearson correlation.

For the *in vivo* study of mouse model corneas, an independent Student’s t-test was used to compare differences between the two groups. A one-way ANOVA with Dunnett’s post-hoc test was used to make comparisons between three or more groups.

## Results

### LC activation levels correlate with SNP preservation

**Fig 1:**
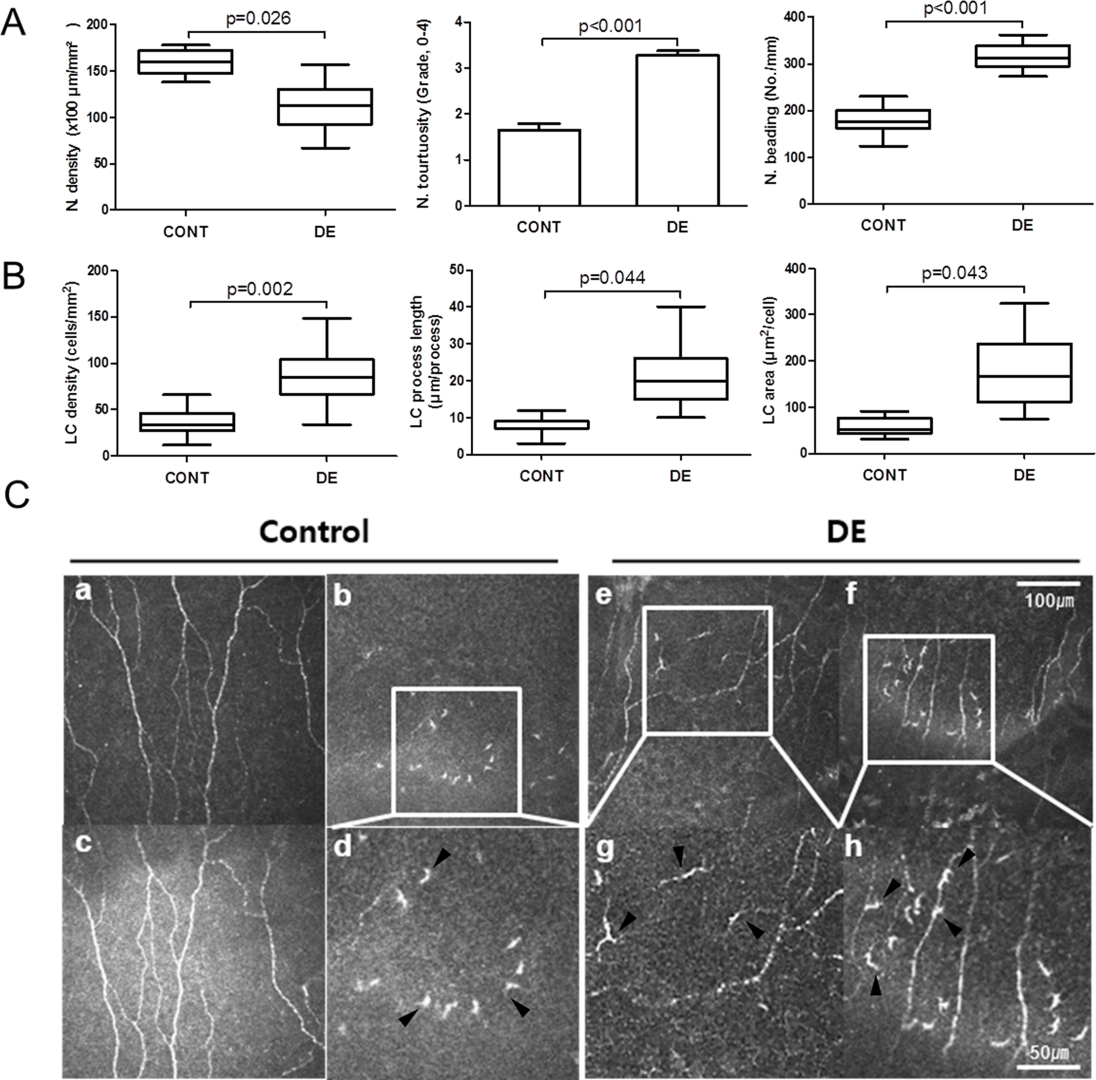
Changes of LCs and the SNP in corneas of patients with DE disease. (A-B) Determination of the mean density and morphological changes of nerves and LCs between the non-DE control (CTL, n = 34) and DE (n = 54). Data are represented as mean ± SD (*: p<0.05 Student t-test, **: p<0.001 Mann–Whitney U test). (C) Representative IVCM image of the SNP and LCs (black arrowheads) from control and DE patients. Figures (Cd), (Cg), and (Ch) are enlarged versions of the squared portions of upper row image.

Mean nerve tortuosity (grade 1.2±0.5 vs. 2.3±0.3, p<0.001) and beading (9.5±3.7 vs. 37.1±8.9 No./mm, p<0.001) were significantly different between the control group patients and DE patients, while mean nerve density was only marginally different between the groups (16040 .4±267.5 vs. 11209.1±315.4 μm/mm^2^, p = 0.026) (Fig 1A). In the DE group, not only was LC density significantly increased, but the average process length and cell area of LCs were also significantly increased (Fig 1B). Representative examples of enlarged LCs with elongated processes are shown on Fig 1Cg (marked with black arrowheads). Interestingly, in some DE patients, LCs had shorter processes and were frequently found near the SNP, contacting nerve leashes (black arrowheads on Fig 1Ch). In the control group, however, all LCs (black arrowheads of Fig 1Cd) had small and rounded cell bodies, and were usually observed separately from the SNP (Fig 1Ca and 1Cc).

**Fig 2:**
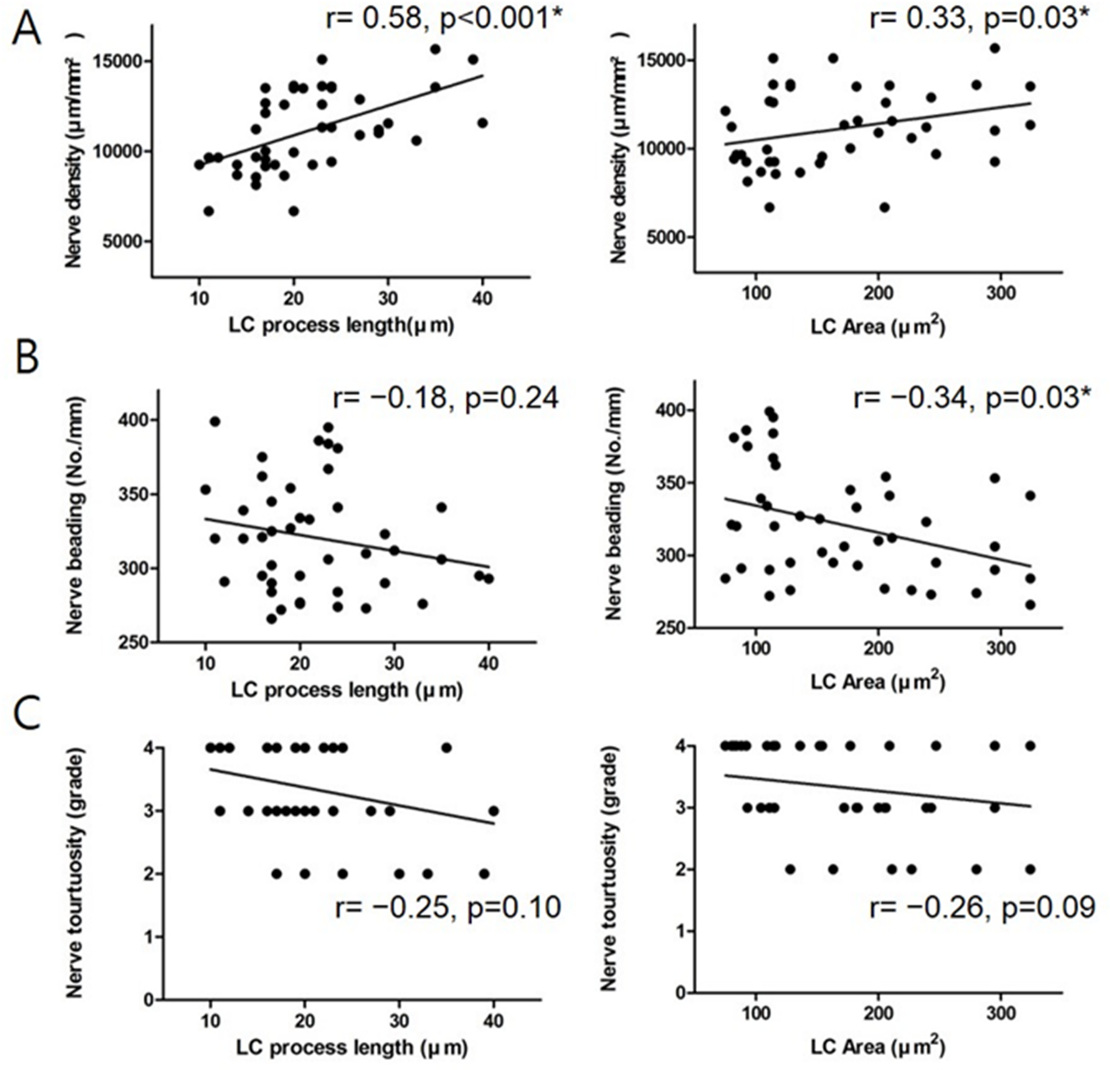
Correlation between LC activation levels and SNP changes in DE patients. (A) Correlation of nerve density with LC process length and with LC area. (B) Correlation of nerve beading with LC process length and with LC area. (C) Correlation of nerve tortuosity with LC process length and with LC area. All relationships were described using Pearson’s correlation coefficient. For schematic demonstration of the correlation, multivariate linear regression analysis was used.

The relationship between LC morphology and SNP damage in DE disease patients was also determined. Changes in LC morphology (such as area and process length) were found to be positively correlated with the SNP density (Fig 2A). Additionally, nerve beading was negatively correlated with LC area and process length (Fig 2B). Nerve tortuosity, however, showed no significant relationship to the LC morphological changes (Fig 2C). LC cell density was poorly correlated with the status of subbasal innervation in the central cornea of DE disease patients (S2 Fig).

### LCs recruited to the ocular surface by DE induction aid in maintaining neural integrity

**Fig 3:**
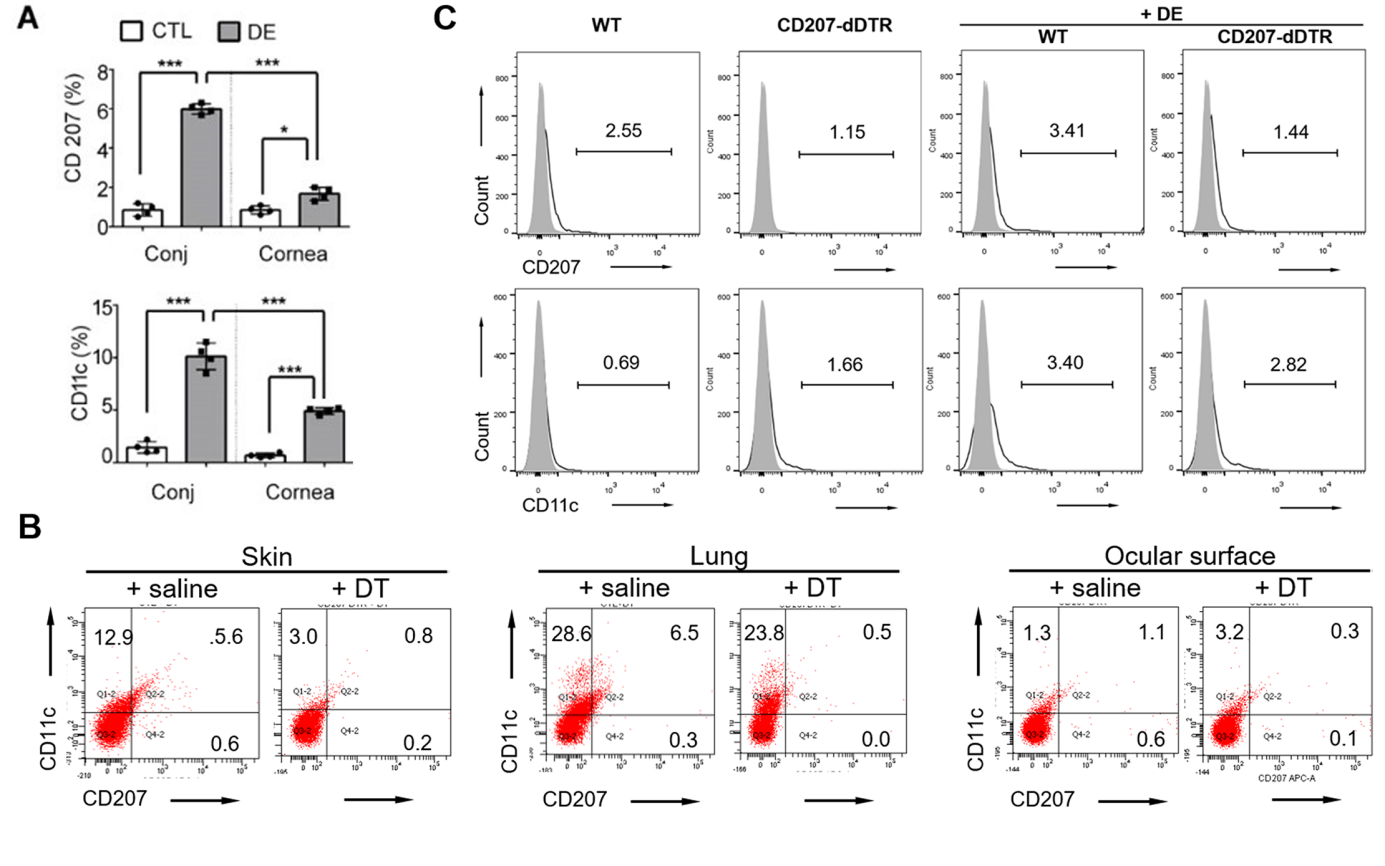
Analysis by flow cytometry of CD207+ and CD11c+ cell recruitment in the cornea and conjunctiva after DE induction. (A) Comparison of CD207+ cell density between the conjunctiva (Conj) and cornea by DE induction. After DE induction, corneal tissues were separated from the conjunctiva for comparison of CD11c and CD207 cell frequencies between the two tissues. A minimum of four mice were included in each group, and the experiment repeated four times. Data are represented as mean ± SD (*: p<0.05, ***: p<0.001 Student t-test). (B) At 10 days post diphtheria toxin injection for CD207-DTR mice, the loss of CD207+ cells in skin, lung, and ocular surface were determined. The experiment was repeated three times and representative flow cytometry data are presented. (C) Comparison of CD207+ and CD11c+ cell population changes in ocular surface by DE induction and CD207-deletion in CD207-DTR mice. At least four mice were included in each group, and the experiment was repeated four times. Representative FACS study data are displayed.

Before DE induction, baseline evaluations revealed that CD207+ cells frequency were 1.1±0.3% in the conjunctiva and 0.9±0.3% in the cornea. After DE induction, a significant increase in both CD207+ cells and CD11c+ cells was observed (Fig 3A). Conjunctival CD207+ cell frequency increased to 5.9±1.2%, a 5.4-fold increase. However, in the cornea, CD207+ cells showed only a 1.67-fold increase over the control (a frequency of 1.5±0.4%). CD11c+ cells increased more than four-fold in both conjunctiva (from 1.5% to 11.4%) and cornea (from 0.8% to 4.7%).

Through the injection of diphtheria toxin into CD207-DTR mice, CD207+ cells were depleted. The loss of CD207+ cells in skin, lung, and ocular surface (conjunctiva and cornea) were confirmed via flow cytometry (Fig 3B). The increase in CD207+ cells found in CD207-DTR+DE control mice was not found in the ocular surface of CD207-dDTR+DE mice. However, as in the WT control group, ocular CD11c+ cells significantly increased in CD207-DTR mice regardless of whether CD207 depletion was induced by diphtheria toxin injection (Fig 3C).

**Fig 4:**
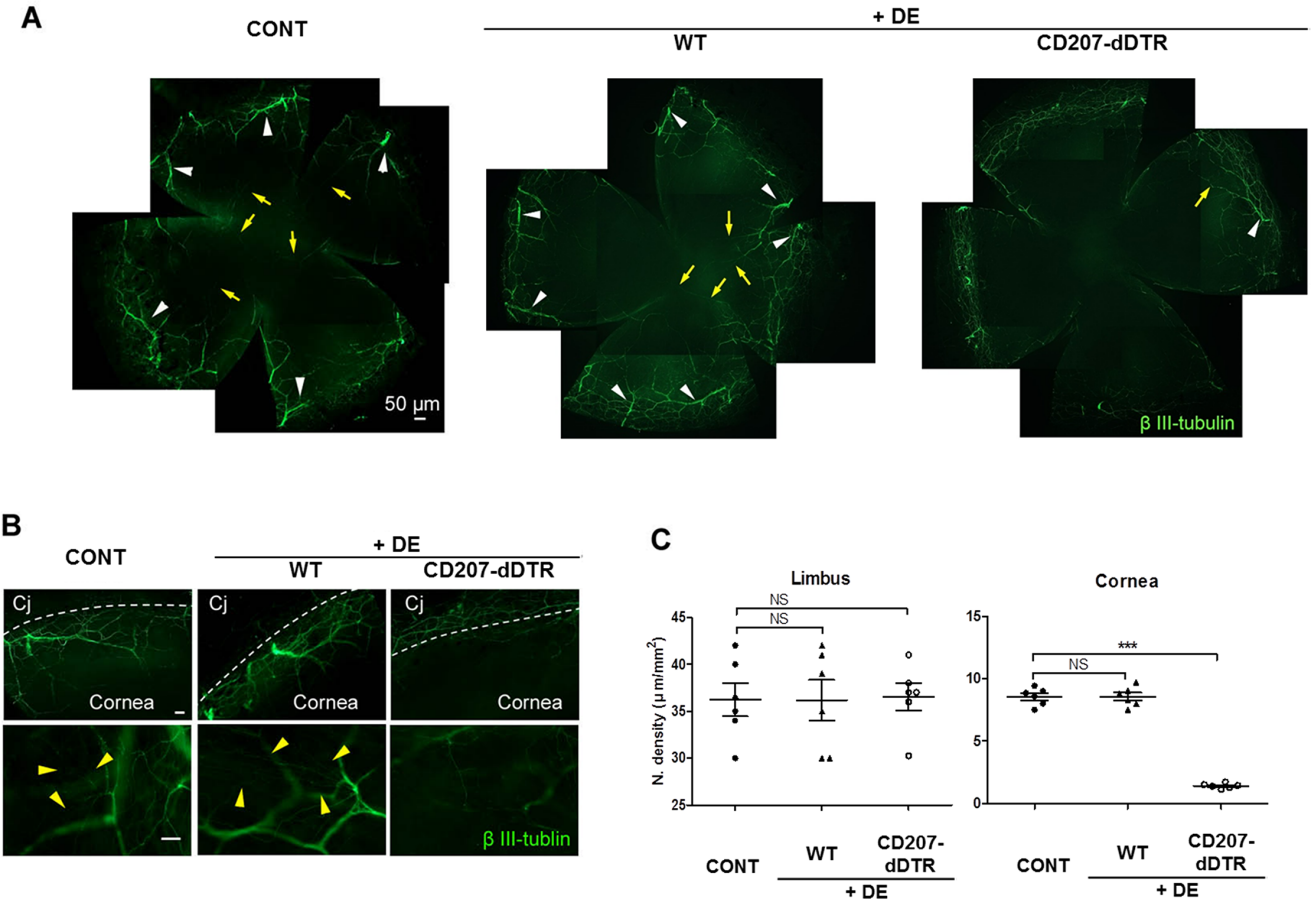
Reduction of corneal nerves in CD207-dDTR+DE mice. (A-C) After 7 days of DE induction of WT mice and CD207-dDTR mice, immunostaining for βIII tubulin (green) on a corneal flap mount was performed and compared with a non-DE induced control (CONT). (A) Low magnification (× 40) photo images. White arrowheads indicate corneal nerves on limbal area with larger diameter (>20 μm) and yellow arrows indicate mid-corneal nerve leashes. (B) High magnification (× 100 upper row and × 200 lower row) images were taken. (C) Limbal and paracentral corneal nerve leashes were compared using ImageJ software in high magnification images between CONT, WT+DE and CD207-dDTR+DE mice. Yellow arrowheads mark the small nerve fibers on the superficial surface of limbal area. At least five mice were included in each group, and nerve length was measured and is represented as mean ± SD. Dashed white line: limbal margin, Cj: conjunctiva, NS: no statistical significance, ***; p<0.0001, One-way ANOVA with Dunnett’s post-hoc test.

The neural integrity of the ocular surface was investigated in WT+DE mice and CD207-dDTR+DE mice. In WT+DE mice, large nerve fibers and fine nerve plexus in the peripheral cornea and limbus (white arrowheads in Fig 4A and yellow arrowheads in Fig 4B) were not significantly reduced by the introduction of DE stress. Moreover, in both conditions (WT and WT+DE), the fine nerve leashes were easily found in central and paracentral cornea (yellow arrows in Fig 4A). However, in CD207-dDTR+DE mice, nerve leashes and large fibers were significantly reduced in the central cornea (yellow arrows Fig 4A and 4C). Large nerve fibers were remarkably thinner in the peripheral cornea (yellow arrowheads Fig 4B), but even with these changes in large fiber diameter, the complexity and total length of the nerve plexus was not reduced in the limbus in CD207-dDTR+DE mice (Fig 4C). The data was not shown here, however, no differences in the major or minor nerve plexus were observed between WT (CONT) and CD207-dDTR mice before DE induction.

### Increased inflammatory cell infiltration and IL-17 response in LC-depleted DE mice

**Fig 5:**
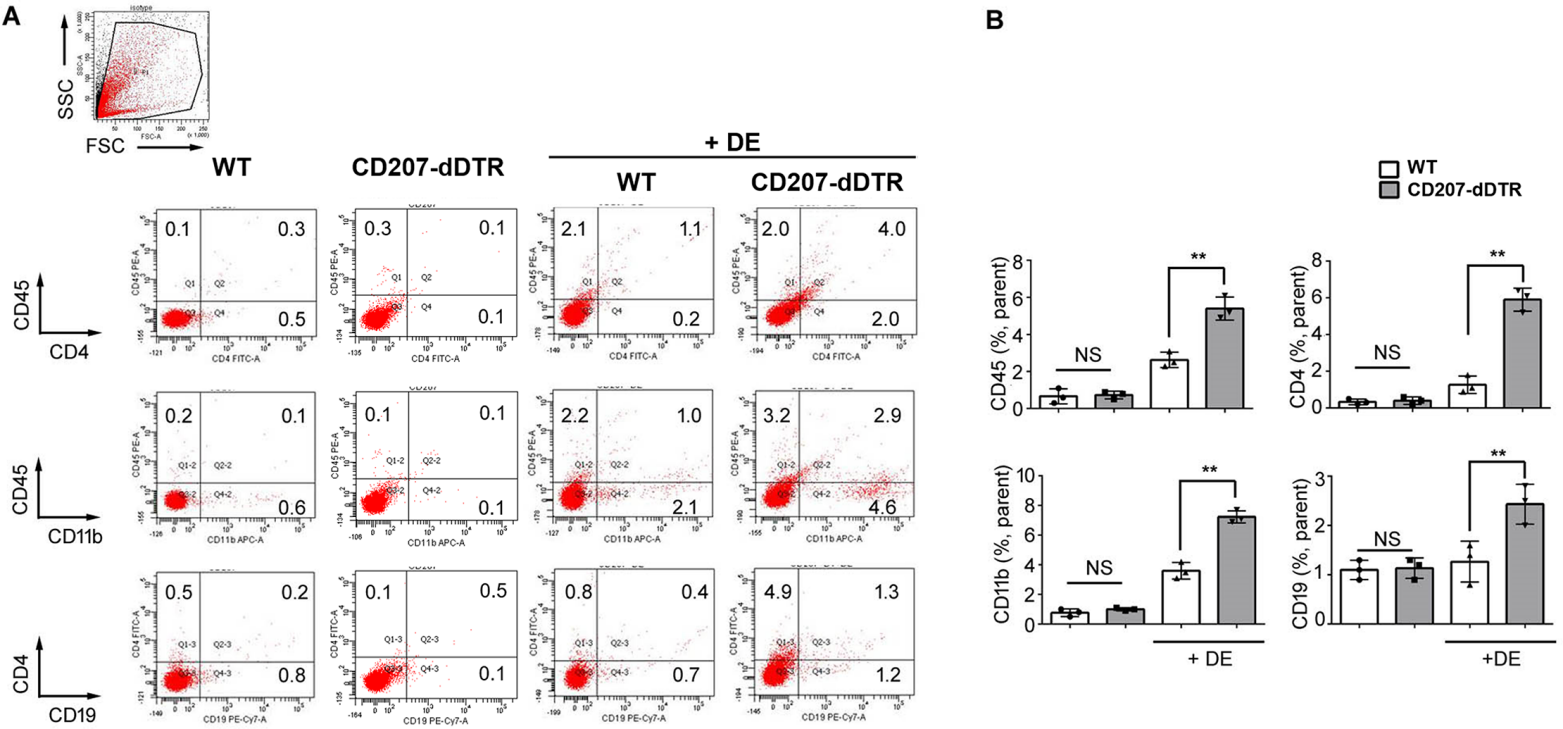
Increased inflammatory cell infiltration in LC-depleted mice by DE induction. (A) Flow cytometry was performed in WT, CD207-dDTR, WT+DE, and CD207-dDTR+DE mice. Cornea samples with limbal tissues were secured and prepared for FACS analysis using anti-CD45-FITC, anti-CD11b-APC, anti-CD4-FITC, and anti-CD19-PE-Cy7 as described in Materials and Methods. At least four mice were included in each group and the experiment was repeated three times. (B) Data were represented as mean ± SD (*: p<0.05, **: p<0.01, ***: p<0.0001 by Student’s t-test).

**Fig 6:**
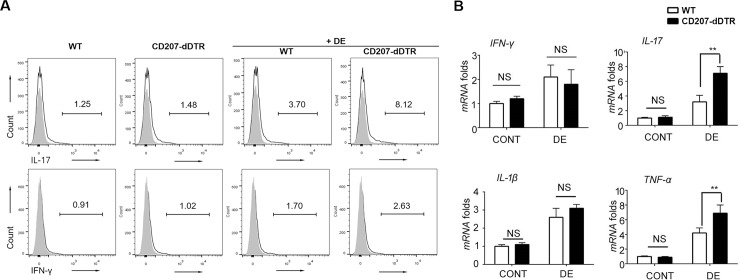
Upregulation of T cell-specific cytokine responses in the ocular surface of LC-depleted DE mice. (A) Flow cytometry was performed in WT mice, CD207-dDTR, WT+DE, and CD207-dDTR+DE mice. Cornea samples with limbal tissues were secured and prepared for FACS analysis using anti-CD207-APC, anti-IFN-γ FITC, and anti-IL-17 PE-Cy7 as described in the Material and Methods section. At least four mice were included in each group and the experiment was repeated three times. Representative FACS data are presented. (B) Quantitative measurement of IL-1β, TNF-α, IFN-γ and IL-17 mRNA in cornea and limbus between WT+DE and CD207-dDTR+DE mice. At least six corneal tissue samples from three mice were included in each group and the experiment was repeated three times. Data are represented as mean ± SD (*: p<0.05, **: p<0.01, ***: p<0.0001 by Student’s t-test).

Prior to DE induction, the numbers of CD45, CD11b, CD4, and CD19 cells in the ocular surface were not significantly different between WT and CD207-dDTR mice (Fig 5A and 5B). However, after DE induction, all four cell types significantly increased in CD207-dDTR mice; notably, CD4+ cell frequencies increased five-fold (1.2% to 6.0%) in CD207-dDTR+DE mice (Fig 5A and 5B). We also measured differences in T cell-specific cytokines, and found that the number of IL-17+ cells significantly increased in CD207-dDTR mice after DE induction (Fig 6A). This increase was also observed at the mRNA level, where an increase of 1.9-fold was noted (Fig 6B). As with IL-17, TNF-α mRNA levels also significantly increased in CD207-dDTR+DE mice (Fig 6B).The number of IFN-γ+ cells was also slightly increased in these LC-depleted DE mice (Fig 6A), but IFN-γ mRNA levels did not change with LC depletion in DE conditions (Fig 6B). IL-1β mRNA levels were also not found to be different between WT+DE and CD207-dDTR+DE mice (Fig 6B).

### Lower levels of neurotrophic factors in the corneas of LC-depleted DE mice

**Fig 7:**
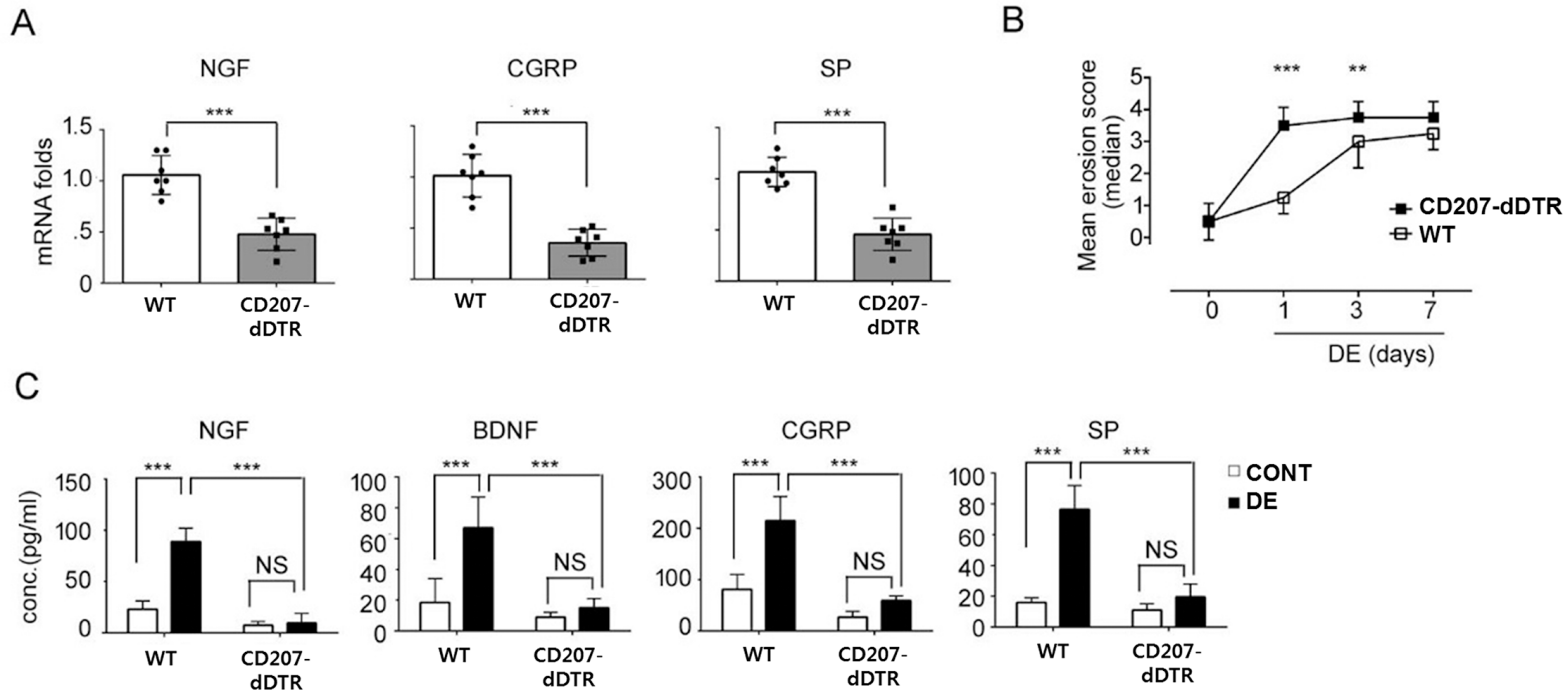
Reduced expression of DE-induced neurotrophic factors by LC depletion. (A) Determination of NGF, CGRP and SP mRNA levels after LC depletion in CD207-DTR mice (CD207-dDTR). (B) Changes in corneal erosion scores for WT+DE and CD207-dDTR+DE (C) NGF, BNGF, CGRP and SP protein levels between WT+DE and CD207-dDTR+DE mice. Data are represented as mean ± SD (*: p<0.05, **: p<0.01, ***: p<0.0001 by Student’s t-test).

Before CD207+ cell depletion, there were no differences in the levels of neurotrophic factors and neurotransmitters between WT and CD207-dDTRmice (data not shown). However, after the depletion of CD207+ cells, ocular surface nerve growth factor (NGF), substance P (SP) and calcitonin gene related peptide (CGRP) levels were significantly reduced, even prior to DE induction (Fig 7A). Corneal erosion scores before DE induction (experiment Day 0) did not differ between WT and CD207-dDTR mice (Fig 7B). With DE induction, the corneal erosion was greater from Day 1 to Day 3 in CD207-dDTR+DE mice (Fig 7B). For CD207-d DTR+DE mice, levels of NGF, CGRP, and brain-derived nerve growth factor (BDNF) did not increase (Fig 7C). However, in WT+DE mice, the neurotrophic factors mentioned above and neuropeptides significantly increased.

## Discussion

### DE-induced LC activation and its role in ocular surface inflammation

Aside from LC cell density, it is known that LC activation levels are also an important factor in LC function and immuno-inflammatory status [36,37]. We found that the depletion of LCs was significantly correlated with the elevation of inflammatory levels in experimental DE status. It was revealed that dermal LCs efficiently induce Th1 cell responses [38], while at the same time presenting soluble antigens to CD8+ T cells [39] and initiating Th17 responses [40]. However, the function of LCs in inflammation has recently been called into question, and there has been a greater attention on their role in tolerance rather than activation of immunity [38], as they have also been found to inhibit contact hypersensitivity [41], viral infection [42], and autoimmune diseases [43,44]. Therefore, LCs may play a suppressive role in certain ocular inflammatory diseases, such as allergic conjunctivitis, corneal allograft rejection, and DE disease.

Intriguingly, known DE-related inflammatory factors are also known LC activators. After exposure to antigens, damage-associated molecular pattern molecules (DAMPs) are stimulated in certain pathological conditions (such as DE disease) and immature LCs acquire an activated phenotype, migrating to lymph nodes for T cell priming [5]. Associated with the LC and lymph node homing process is matrix metalloproteinase 9 (MMP9), a well-known matrix protein also involved in cell process elongation, lymphatic invasion, and matrix degradation to facilitate LC migration [45]. Moreover, prostaglandin E2 (PGE2) may play a critical role in MMP9 expression in addition to its role in LC activation [35,46]. Since MMP9 and PGE2 are essential components for DE-induced inflammation, the functional role of these factors on LC activation should be investigated further.

For the first time, we revealed in DE patients that the activation level of LCs which can be determined with their morphological parameters was negatively related with the loss and deformation of subbasal nerve. *In vivo* studies of mouse corneas were included in addition to studies of human corneas to improve the reliability of the conclusions. We confirmed that LCs (CD207+ cells) on the ocular surface could be specifically removed with diphtheria toxin injection in the CD207-dDTR mouse model, without any changes in CD11c population. No increase in ocular surface CD207+ cells in CD207-dDTR+DE mice was found, whereas CD11c+ cells were significantly increased in CD207-dDTR mice (similar to the increase seen in WT mice). Increased CD4+ T cell infiltration and upregulation of inflammatory chemokines and cytokines in CD207-dDTR+DE mice were observed compared to WT+DE mice. Therefore, we suggest that CD207+ cells have a negative role in regulating the ocular surface immuno-inflammation of DE disease.

However, we could only analyze a small part of the ocular surface in humans due to limitations of IVCM methodology. Because it is well known that LCs are more abundant in the conjunctiva and peripheral cornea than in the central cornea, we examined the central corneal only to analyze the relationship between nerves and LCs. The gradual reduction of LC density from the peripheral area to the central cornea was confirmed [47], and the increased LC population in the central cornea was proportionally correlated with DC density of the peripheral cornea, as was noted in previous studies[16,48]. In addition, LCs visualized using IVCM may not be authentic in an *ex vivo* setting, as there are phenotypically different CD11c+ Langerin+ populations in the epithelium (CD11b^low^CD103^low^) and in the stroma (CD11b+CD103^low^) [16]. Immunostaining for specific markers for each cell type is necessary to precisely document different phenotypes of Langerin+ populations in human corneas. As we only used IVCM for this study, we were unable to clearly document whether the intraepithelial DCs were authentic LCs.

### The role of LCs in neural integrity during pathogenesis of DE

Using the CD207-dDTR+DE mouse, we showed that the loss of LCs in the corneal epithelium resulted in significant downregulation of neurotrophic factors, along with the loss of paracentral corneal nerves and aggravated corneal erosion. These results indicate the importance of LCs for maintaining the neural integrity of the ocular surface and the prevention of corneal epitheliopathy during DE disease.

Additionally, previous studies report that innervation affects the activation status of LCs under normal and pathological conditions. LCs in the skin were shown to be closely related with cutaneous nerves while targeting them with secretion of nerve products [49,50]. Additionally, it has been reported that neurotrophic factors and neurotransmitters also regulate LC function [29,50]. Since neurotransmitters (e.g. vasoactive intestinal polypeptide (VIP), pituitary adenylate cyclase-activating peptide (PACAP), CGRP, and SP) can be released specifically from the main nerve fibers in the cornea (ref above, 51, 55), taken together, these findings suggest that LCs and corneal nerves may communicate closely and reciprocally effect on their function for maintaining the ocular surface homeostasis.

We could not clearly identify which neurotransmitters are critical for LC density and activation, nor which neurotrophic factors are required for maintaining functional neural integrity and LC populations in the DE pathophysiology. Although we demonstrated that mRNA levels for NGF and other neurotrophic factors were markedly reduced in the LC-depleted cornea under DE conditions, these results may not necessarily imply that LCs are the direct source of these neurotrophic factors. Considering that some peptides derived from corneal epithelium [51] and keratocytes [52] also have neurotrophic activity, a possible explanation is that LCs regulate neurotrophic factor synthesis in the corneal epithelium, conceivably through a release of cytokine-like factors.

In conclusion, we have found that LCs are essential for maintaining subbasal nerve health and for regulating ocular surface inflammation in DE disease. Future studies are needed to investigate the precise mechanisms of LCs in ocular surface inflammation and innervation in DE disease. Additionally, it will be necessary to identify which specific cytokines recruit LCs and maintain their activity in the DE condition. The role of DE-induced LCs in the activation of T cell population will also need to be determined and compared with other subtypes of ocular surface DCs.

## Supporting information

S1 Fig Schematic protocols of the in vivo experiment. WT = Wild-type; DE = dry eye; CTL = Control; DT = Diphtheria toxin; CD207-DTR = CD207-diphtheria toxin receptor; CD207-dDTR = CD207-depleted DTR.
(TIF)

S2 Fig Correlation of LC density and intraepithelial innervation status (nerve density, beading, and tortuosity). Pearson’s correlation analysis was used. For schematic demonstration of the correlation, multivariate linear regression analysis was used.
(TIF)

S1 Table Primers used for qRT-PCR analysis of proinflammatory cytokine and neurotrophic factor/neurotransmitter expression. (PDF)
