# Supplementary figures and images for: Langerhans cells prevent subbasal nerve damage and upregulate neurotrophic factors in dry eye disease
Source: PLoS One. 2017 Apr 25;12(4):e0176153. doi: 10.1371/journal.pone.0176153 (PMC5404869; doi:10.1371/journal.pone.0176153)

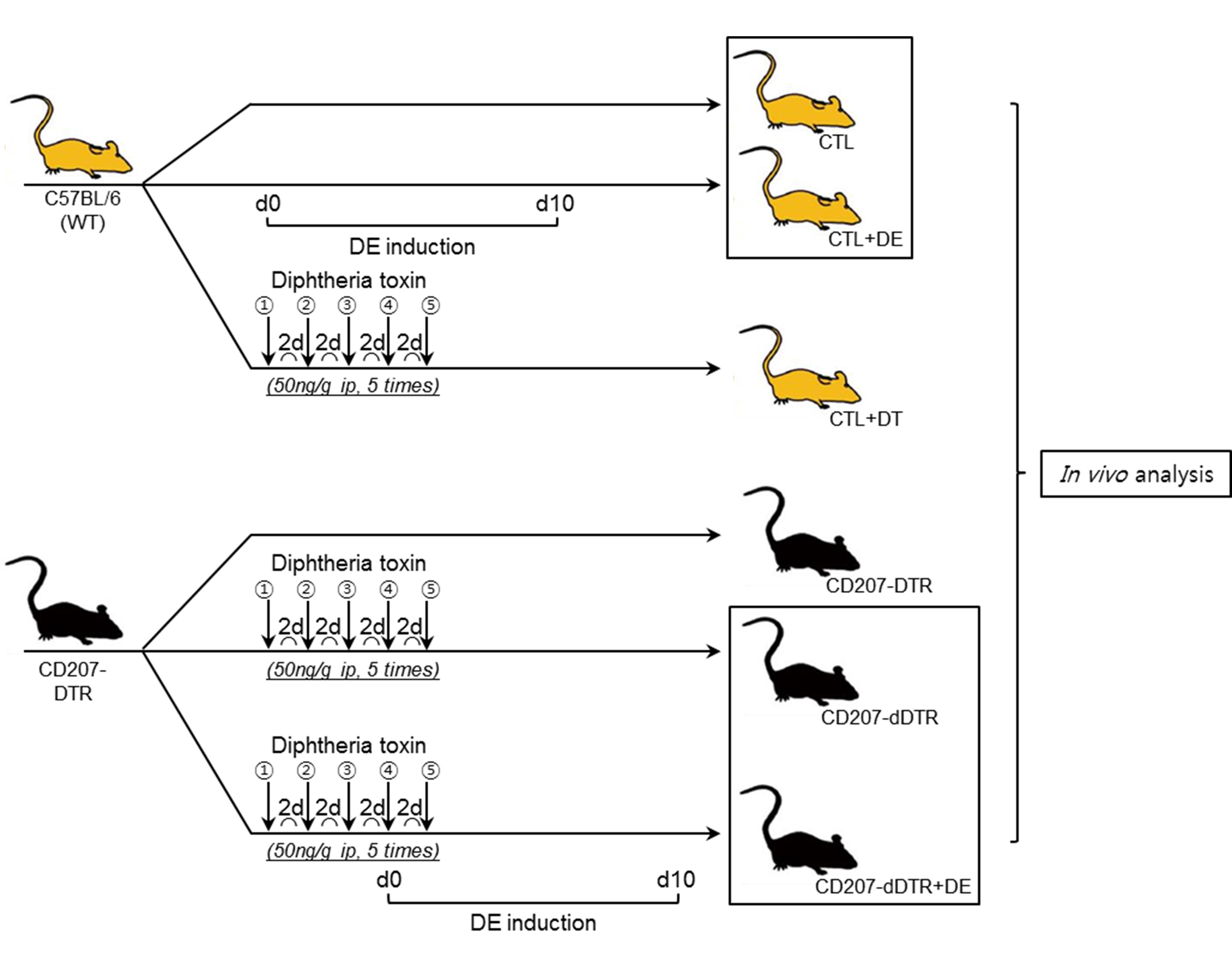

Supplement: S1 Fig — WT = Wild-type; DE = dry eye; CTL = Control; DT = Diphtheria toxin; CD207-DTR = CD207-diphtheria toxin receptor; CD207-dDTR = CD207-depleted DTR. (TIF) [file pone.0176153.s001.tif]

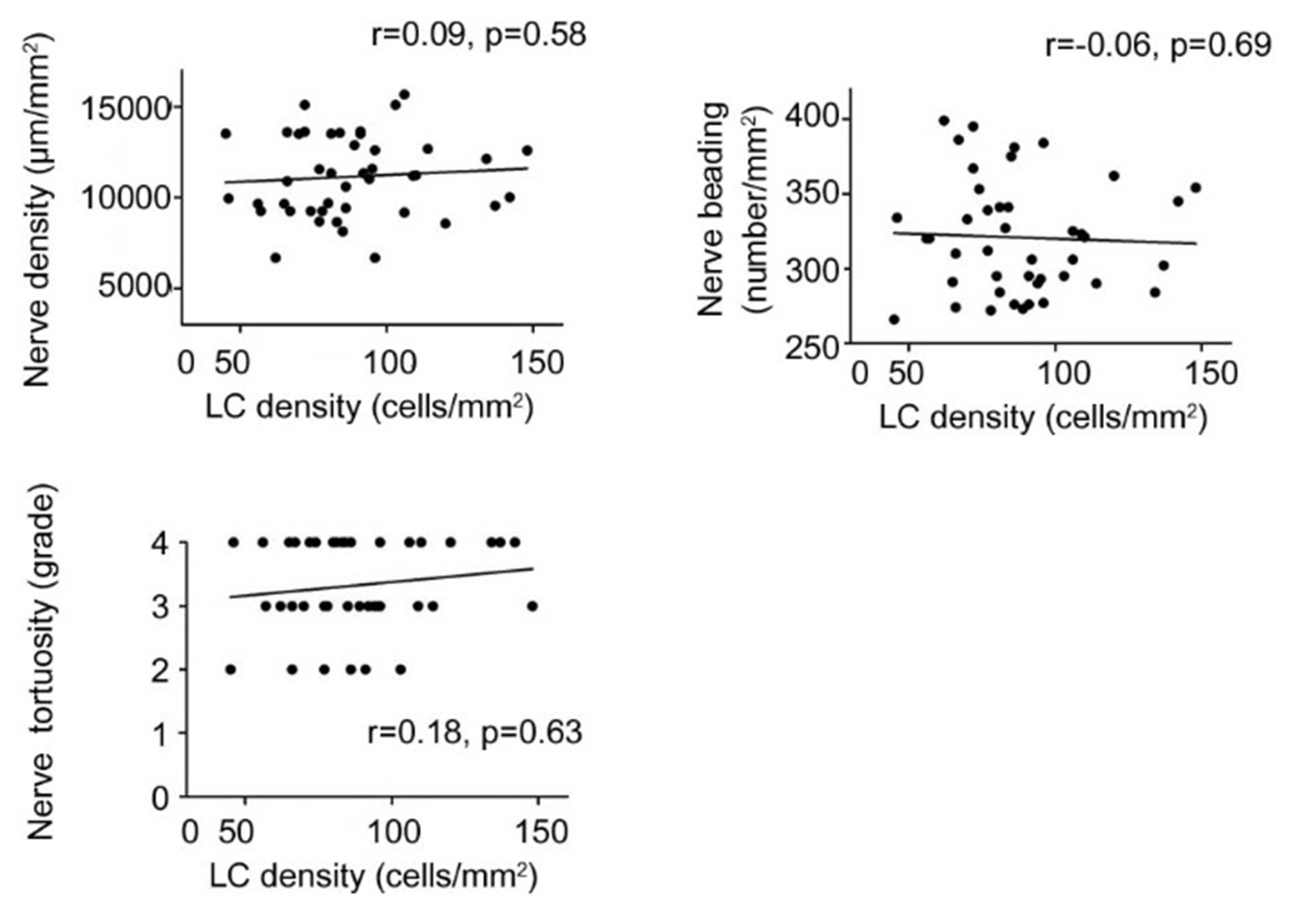

Supplement: S2 Fig — Pearson’s correlation analysis was used. For schematic demonstration of the correlation, multivariate linear regression analysis was used. (TIF) [file pone.0176153.s002.tif]
