# Supplementary material for: Langerhans cells prevent subbasal nerve damage and upregulate neurotrophic factors in dry eye disease
Source: PLoS One. 2017 Apr 25;12(4):e0176153. doi: 10.1371/journal.pone.0176153 (PMC5404869; doi:10.1371/journal.pone.0176153)
Supplement: S1 Table — (PDF) [file pone.0176153.s003.pdf]

S1 Table. Primers used for qRT-PCR analysis of proinflammatory cytokine and neurotrophic factor/neurotransmitter expression.

Target gene	Sequence	Reference gene
IFN-γ	F: ACA AAG ATG GCA GAG CAC GA<br>R: TCC ACC AAC ATG TGC GGT TT	NM_008337
IL-6	F: TGG CTA AGG ACC AAG ACC AT<br>R: TAA CGC ACT AGG TTT GCC GA	NM_031168
IL-1β	F: AAG GGC TGC TTC CAA ACC TTT GAC<br>R: ATA CTG CCT GCC TGA AGC TCT TGT	NM_008361
IL-17	F: TCC ACC GCA ATG AAG ACC CTG ATA<br>R: ACC AGC ATC TTC TCG ACC CTG AAA	NM_010552
Nerve growth factor (NGF)	F: GTG TGT GGG TTG GAG ATA AG<br>R: AGT ACT GTC TGA ATA CAC TGT TG	NM_001112698
Brain-derived nerve growth factor (BNGF)	F: GAG CGT GTG TGA CAG TAT TAG<br>R: CTT TGG ATA CCG GGA CTT TC	NM_001048139
Substance P (SP)	F: CCT CAG CAG TTC TTT GGA TTA<br>R: CTG GCC ATG TCC ATA AAG AG	NM_009311
Neuropeptide Y (NPY)	F: ATC TCT GCT CGT GTG TTT G<br>R: GCA GAG CGG AGT AGT ATC T	NM_023456
Calcitonin gene related peptide (CGRP)	F: CAC TGG TGC AGG ACT ATA TG<br>R: TCC CAC ACC GCT TAG AT	NM_001033954
Neurotrophin (NT)-3	F: AGA GCT ACT ACG GCA ACA<br>R: GCC CAC ATA ATC CTC CAT TAG	NM_001164034
NT-4/5	F: CTC CTG TTC TCT CCT CCT TT<br>R: GAC AAG AGG TCC CAC TCA	NM_198190
GAPDH	F: TCA ACA GCA ACT CCC ACT CTT CCA<br>R: ACC CTG TTG CTG TAG CCG TAT TCA	NM_001289726

INF- *γ* = interferon- *γ*; IL = interleukin; GAPDH = glyceraldehyde-3-phosphate dehydrogenase.
